# Changes in cytokine responses to TB antigens ESAT-6, CFP-10 and TB 7.7 and inflammatory markers in peripheral blood during therapy

**DOI:** 10.1038/s41598-018-19523-7

**Published:** 2018-01-18

**Authors:** Ah Young Leem, Joo Han Song, Eun Hye Lee, Hyejon Lee, Bora Sim, Song Yee Kim, Kyung Soo Chung, Eun Young Kim, Ji Ye Jung, Moo Suk Park, Young Sam Kim, Joon Chang, Young Ae Kang

**Affiliations:** 1 Division of Pulmonology, Department of Internal Medicine, Institute of Chest Disease, Severance Hospital, Yonsei University College of Medicine, 50-1 Yonsei-ro, Seodaemun-gu, Seoul, 120-752 Republic of Korea; 2 Department of Microbiology and Institute of Immunology and Immunological Disease, Yonsei University College of Medicine, Seoul, Republic of Korea

## Abstract

Multiple cytokines and inflammatory markers control TB infection. We aimed to investigate the changes in multiple cytokines and inflammatory markers in active TB patients following anti-TB drug therapy. Twenty-nine patients with active TB were recruited prospectively between December 2010 and July 2017. Blood samples were collected before (T_0_), after 2 months (T_2_), and at the end of anti-TB treatment (T_end_). We measured the levels of Interferon (IFN)-γ, interleukin (IL)-2, IL-12, IL-10, IL-13 and tumor necrosis factor (TNF)-α in supernatants collected from the QuantiFERON-TB Gold In-Tube assay (QFT-GIT), as well as the WBC, neutrophil, platelet count and neutrophil to lymphocyte ratio (NLR) in whole blood. Compared with baseline levels, WBC, neutrophil, and platelet counts were significantly lower following treatment. In addition, the NLR after treatment significantly decreased compared with baseline, whereas the IL-2/IFN-γ ratio increased after treatment. In conclusion, the levels of IL-2/IFN-γ ratios in the supernatant and the NLR might be useful biomarkers to evaluate the effectiveness of drug therapy in active TB patients.

## Introduction

Tuberculosis remains a major cause of death and morbidity worldwide, responsible for an estimated 1.7 million deaths each year^1^. Prolonged treatment is required for adequate therapy, resulting in significant costs and medical resources.

The ability to monitor the response of TB to therapy and confirm adequate treatment would constitute a major advancement. There have been efforts to develop biomarkers for the diagnosis and treatment of TB. Identifying biomarkers of treatment success might provide insight into surrogate markers of protective immunity against TB. Cell-mediated immunity plays an important role in human host defenses against TB^2^. The essential role of T cells in protection against TB infection has been well-documented^2^. IFN-γ, a cytokine produced by T cells, is used to diagnose TB infection^3^. Other cytokines, including TNF-α^4^, IL-12^5^, IL-13^6^, and IL-2^7^ are essential in controlling TB infection. Furthermore, IFN-γ and IL-2 profiles have been associated with antigenic load and treatment in active TB^8^.

Although several studies have evaluated IFN-γ to monitor the response to anti-TB treatment, these studies yielded conflicting results, with IFN-γ responses decreasing^9–11^, increasing^12^, or remaining not significantly changed^13,14^ in response to treatment. An *in vitro* IFN- γ immune diagnostic assay for active TB disease, the novelty of which consists of the use of multiepitopic region of difference (RD)1 peptides selected by computational analysis was reported^15–17^. In the study of Goletti *et al*., the response to selected RD1 peptides was associated with TB disease in HIV-infected individuals, and decreased after successful therapy^9^. Several exploratory studies have evaluated the diagnostic potential of cytokine biomarkers other than IFN-γ for monitoring anti-TB treatment such as TNF-α^18^, or IFN- γ /TNF-α^19^, or IFN- γ inducible protein (IP)-10^20^.

However, no single cytokine or combination of cytokines has shown a strong correlation with treatment success. To date, relatively few studies have evaluated the usefulness of multiple cytokines in monitoring the response to anti-TB treatment^21,22^. Previously, we examined multiple cytokines, including IFN-γ and IL-2, to determine the TB infection status and assess mycobacterial loads among smear-positive, smear-negative, and latent TB infection^23^. In the current study, we investigated the changes in multiple cytokines and inflammatory markers in active TB patients following anti-TB drug therapy.

## Materials and Methods

### Study population

Patients with active pulmonary TB were recruited prospectively between December 2010 and July 2017 at Severance Hospital, a tertiary referral hospital in Seoul, South Korea, after approval of the study protocol by the Ethics Review Committee. The patients who could not be followed for at least 6 months, those with HIV infection, end-stage renal disease, or leukemia/lymphoma, and those undergoing immunosuppressive therapy within 3 months of enrollment were excluded. After providing informed consent, each patient completed a set of questionnaires pertaining to demographics, history of TB, and cigarette use. The QFT-GIT was performed in each patient T_0_, T_2_, and T_end_. We also measured the levels of IFN-γ, IL-2, IL-12 (p40), IL-10, IL-13, and TNF-α in the supernatant obtained from the QFT-GIT, as well as the white blood cell (WBC) count, neutrophil count, lymphocyte count, platelet count, red cell distribution width (RDW), mean platelet volume, neutrophil to lymphocyte ratio (NLR), the ratio of monocytes and lymphocytes (MLR) and platelet to lymphocyte ratio (PLR) in all patients at T_0_, T_2_, and T_end_.

All participants provided a written, informed consent for the collection of samples and subsequent analysis. All the individuals participating in this study were over 18 years old. All methods were carried out in accordance with relevant guidelines and regulations. The study protocol was reviewed and approved by the Institutional Review Board of Yonsei University Health Service, Severance Hospital (4-2013-0803).

### Diagnosis and TB treatment

The diagnosis of active pulmonary TB was made based on clinical, radiological, microbiological, and pathological data. Active pulmonary TB was confirmed in TB cultures derived from respiratory specimens or by the presence of caseating granulomas in lung tissue. One patient who was highly suspected of having active TB but had a negative mycobacterial culture showed good clinical and radiographic responses to TB treatment and therefore was also included. Treatment was based on the Korean Guidelines for Tuberculosis 2011. The standard regimen of rifampicin (10 mg/kg body weight [BW]/day), isoniazid (5 mg/kg BW/day), ethambutol (15–25 mg/kg BW/day), and pyrazinamide (15–30 mg/kg BW/day) was used. The duration of treatment was at least 6 months, depending on the risk of recurrence, the clinical response to treatment, and drug intolerance. Patients who had positive mycobacterial culture at the end of the intensive phase (after two months of the treatment) with cavity lesion in their chest radiograph were considered as having high risk of relapse.

### QFT-GIT assay

IFN-γ release assays were performed using the QFT-GIT assay kit according to the manufacturer’s instructions. Whole blood (1 mL) was collected in each of three tubes precoated with saline (nil control, Nil), TB-specific antigen (TB Ag; ESAT-6, CFP-10, and TB7.7), or mitogen and incubated for 20 h at 37 °C within 8 h of blood sampling. The plasma supernatant was collected after centrifugation and stored at −20 °C until assayed for IFN-γ using the QFT GOLD ELISA. Results were calculated using the manufacturer’s QFT-GIT software.

### Multiplex analysis of cytokine production

The levels of multiple cytokines, IFN-γ, IL-2, IL-12(p40), IL-10, IL-13, and TNF-α, in the supernatants obtained from the QFT-GIT assays were measured using the commercial MILLIPLEX® MAP human cytokine/chemokine kit (HCYTOMAG-60K-06, Millipore, Billerica, MA, USA) with fluorescently labeled microsphere beads and a Luminex reader. The cytokine levels were measured in the QFT-GIT supernatants at T_0_, T_2_, and T_end_. The undiluted supernatants were used in this study. The minimum detectable concentrations were 0.8 pg/mL for IFN-γ, 1.0 pg/mL for IL-2, 7.4 pg/mL for IL-12(p40), 1.1 pg/mL for IL-10, 1.3 pg/mL for IL-13, and 0.8 pg/mL of TNF-α according to the protocol. The value under minimum detectable concentrations were considered as zero.

### Statistical analysis

The quantitative data for cytokines were expressed as numbers with percentage in brackets or as medians with the interquartile range (IQR) in brackets. Cytokine levels were analyzed using repeated measures analysis of variance to compare the expression of each cytokine among three groups. The Wilcoxon signed rank test was used to compare cytokine expression between two related groups. The GraphPad Prism program version 4.0 (GraphPad software, San Diego, CA, USA) was used to create the graphics. A two-tailed p-value < 0.05 was considered significant. All statistical analyses were performed using SPSS version 15.0 (SPSS Inc., Chicago, IL, USA).

## Results

### Baseline patient characteristics

The clinical characteristics of the study patients, including the extent of the disease in chest CT, acid-fast bacilli (AFB) smear and culture positivity, and profiles obtained from the blood tests and QFT-GIT assay, are summarized in Table 1. Twenty-two (75.9%) patients were culture-positive on sputum specimens, and all 22 achieved sputum conversion. The median (IQR) level of the IFN-γ concentration obtained in the TB Ag tube in all patients was 9.47 (4.51–10.0) IU (international units)/mL.

**Table 1:** Demographics and clinical data.

	Total (N = 29)
**Age**, **median** (**IQR**)	27 (25–38)
**Male**, **n** (**%**)	16 (55.1)
**BMI**, **median** (**IQR**)	20.3 (19.2–21.9)
**Symptom**, **n** (**%**)	
cough	13 (44.8)
sputum	15 (51.7)
fever	4 (13.8)
weight loss	3 (10.3)
fatigue	9 (31.0)
chest pain	7 (24.1)
dyspnea	3 (10.3)
hemoptysis	3 (10.3)
others	1 (3.4)
**Chest CT finding**, **n** (**%**)	
centrilobular	9 (31.0)
consolidation	11 (37.9)
cavity + centrilobular	8 (27.6)
mass	1 (3.4)
**Extent of the disease in chest CT**, **the number of lobes** (**%**)	
One lobe	20 (69.0)
2–3 lobes	9 (31.0)
>3 lobes	0
**Blood tests**, **median** (**IQR**)	
WBC count (/µL)	6990.0 (5750.0–8380.0)
Hemoglobin level (g/dL)	13.8 (12.3–14.9)
Platelet count (×10^3^/µL)	292.0 (251.0–387.0)
**Sputum exam**, **n** (**%**)	
AFB smear, positive	3 (10.3)
TB culture, positive	22 (75.9)
sputum conversion	22 (100.0)
**QFT-GIT assay**, **median** (**IQR**)	
Nil	0.21 (0.13–0.28)
TB Ag	9.47 (4.51–10.00)
mitogen	10.00 (10.00–10.00)
TB Ag-Nil	8.6 (4.2–10.00)
**Extrapulmonary TB**, **n** (**%**)	1 (3.4)

### Multiple cytokine responses to TB –specific antigens before, after 2 months, and after treatment of active pulmonary TB

**Figure 1:**
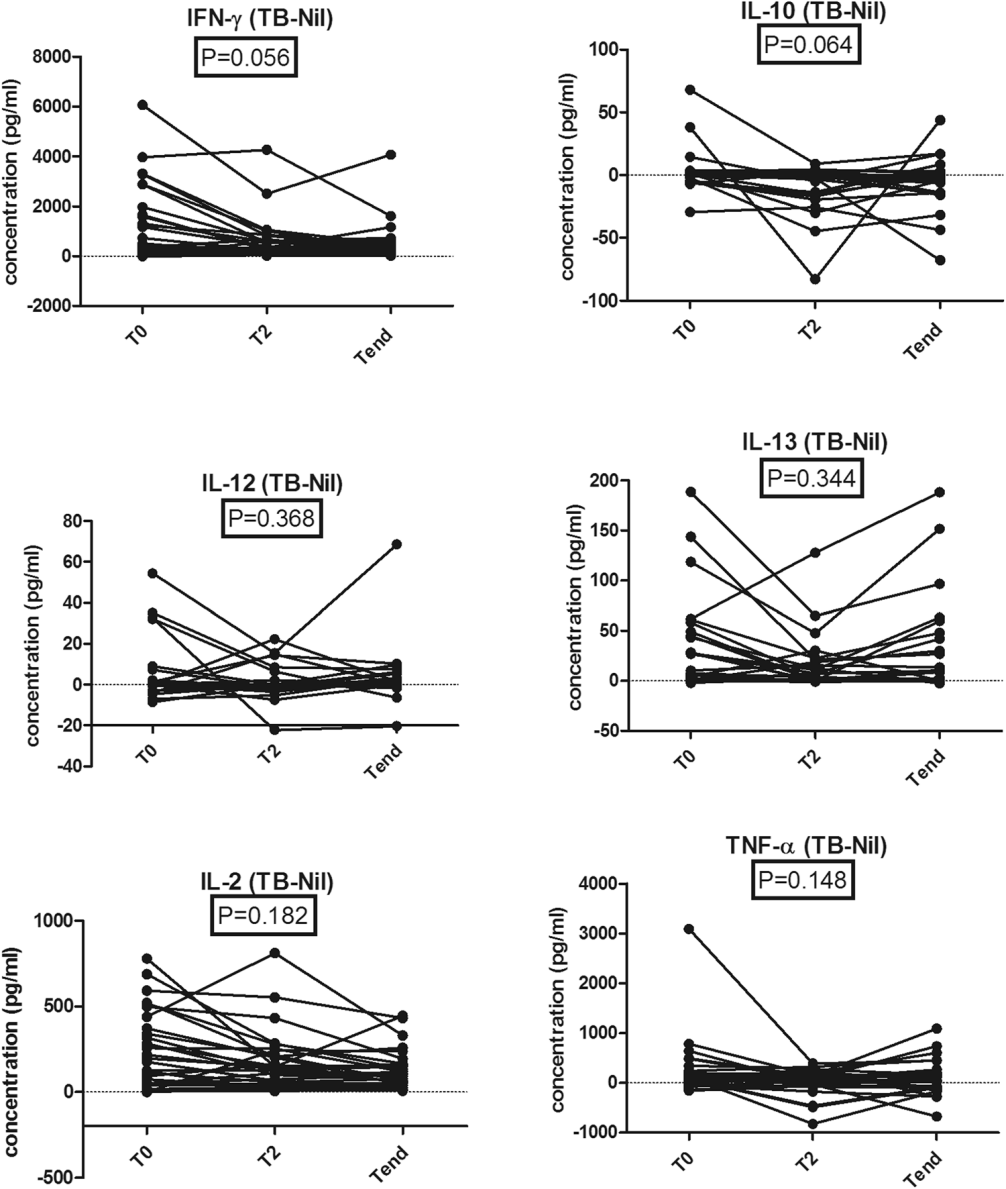
The changes of levels of IFN-γ, IL-10, IL-12, IL-13, IL-2 and TNF-α in supernatants of QFT-GIT before (T_0_), after 2 months (T_2_), and at the end of anti-TB treatment (T_end_).

We measured the levels of multiple cytokines at the major time points of anti-TB treatment for active pulmonary TB (Fig. 1). The response was calculated by subtracting the level in the Nil sample from that in the TB Ag sample. The mean changes from T_0_ to T_end_ of IFN-γ, IL-10, IL-12, IL-13, IL-2 and TNF-α were −770.21 pg/ml (95% CI, −1150.72 to −389.69), −7.52 pg/ml (95% CI, −14.05 to −1.00), −1.80 pg/ml (95% CI, −7.17 to 3.57), −5.09 pg/ml (95% CI, −21.39 to 11.20), −118.37 pg/ml (95% CI, −180.69 to −56.05), and −176.36 pg/ml (95% CI, −389.22 to 36.48). The proportions of subjects that achieved these change for IFN-γ, IL-10, IL-12, IL-13, IL-2, and TNF-α were 65.5%, 68.9%, 55.1%, 62.0%, 58.6%, and 62.0%.

**Figure 2:**
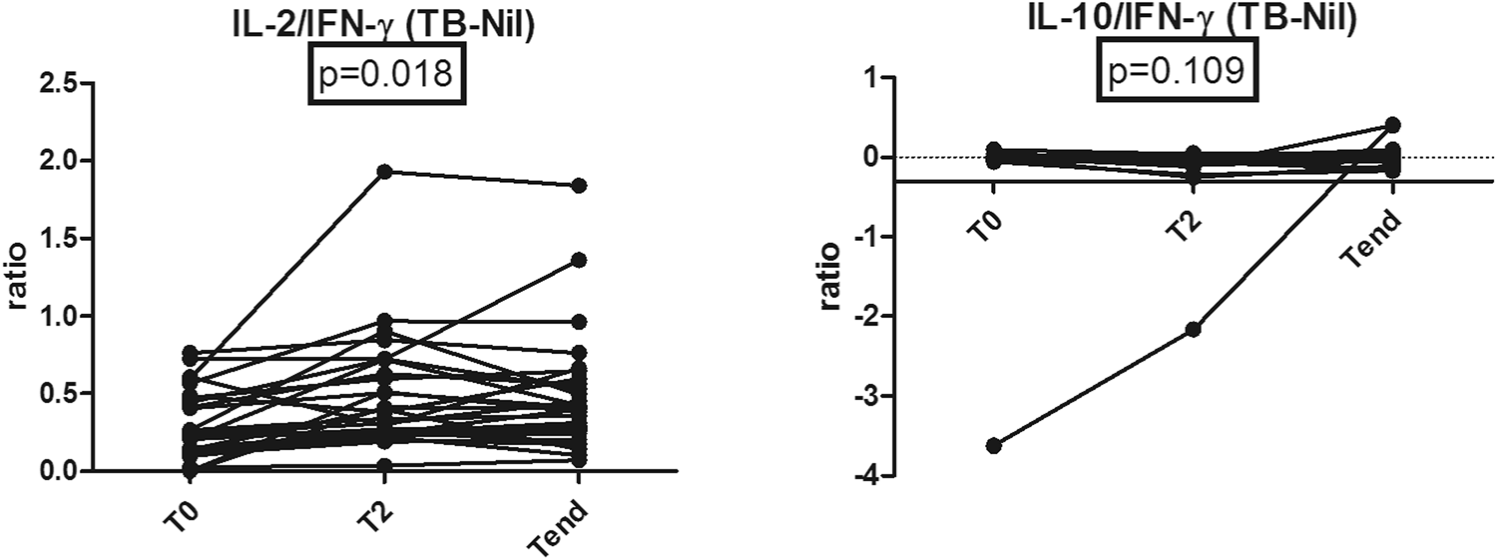
The changes of IL-2/IFN-γ and IL-10/IFN-γ ratios in supernatant of QFT-GIT during anti-TB treatment.

The median decreases in IFN-γ, IL-10, IL-12, IL-13, IL-2, and TNF-α levels were not significant (Fig. 1). In addition the mean changes from T_0_ to T_end_ of IL-2/IFN-γ ratio and IL-10/IFN-γ were 0.19 (95% CI, 0.07 to 0.30) and 0.12 (95% CI, −0.15 to 0.41). The proportions of subjects that achieved these change for IL-2/IFN-γ ratio and IL-10/IFN-γ were 24.1% and 27.5%. The IL-2/IFN-γ ratio measured in the supernatant after treatment was significantly increased compared with baseline (*P* = 0.018), whereas the median decrease in IL-10/IFN-γ ratio not significant (Fig. 2).

### Inflammatory markers in peripheral blood before, at 2 months, and after treatment of active pulmonary TB

**Figure 3:**
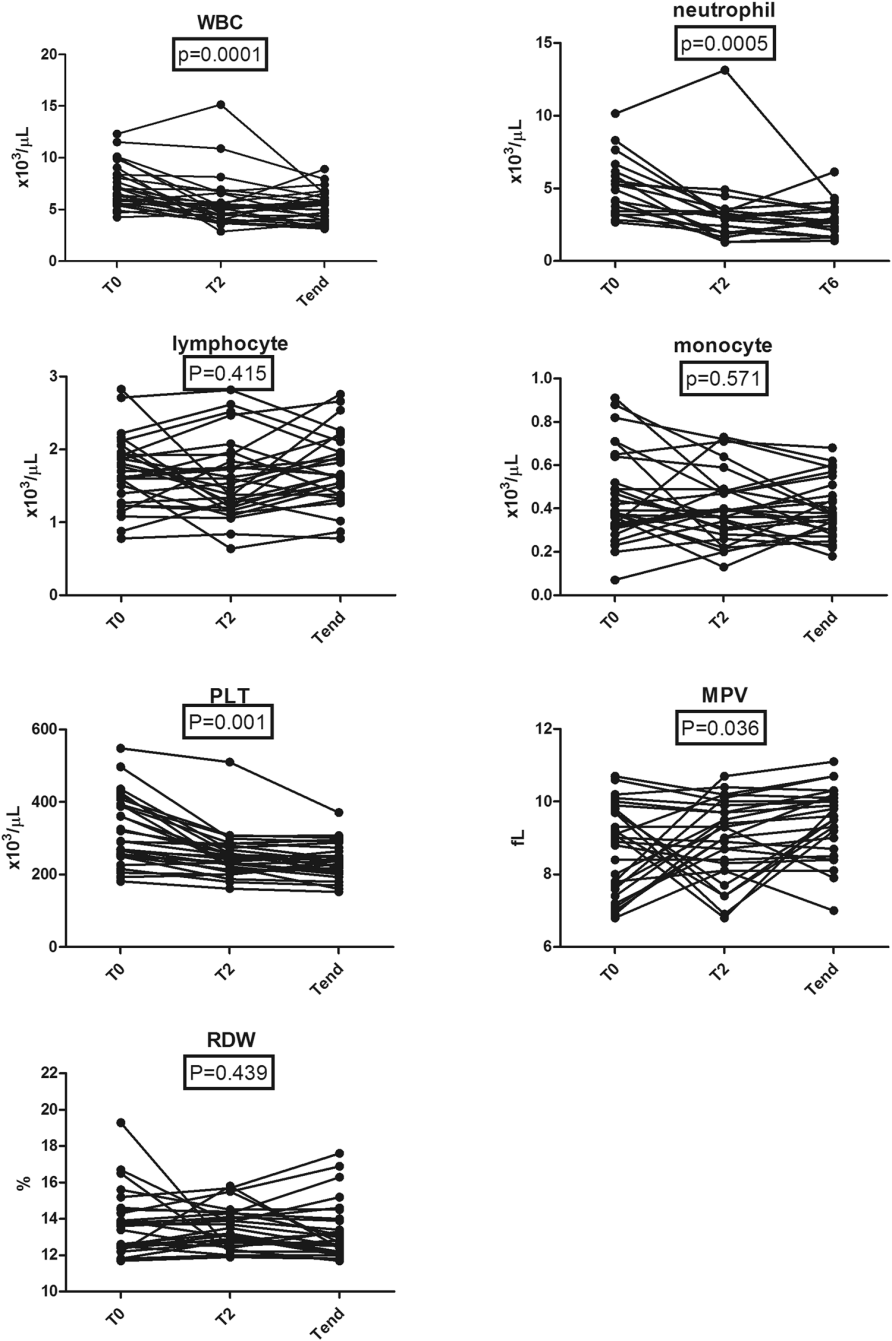
The changes of serum inflammatory markers during anti-TB treatment.

**Figure 4:**
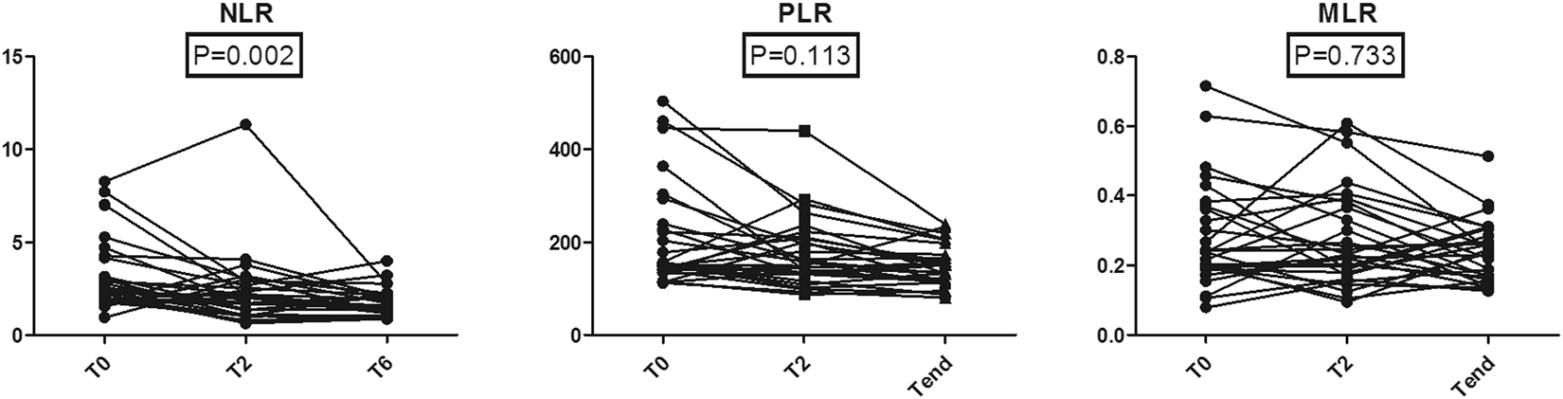
The changes of neutrophil to lymphocyte, platelet to lymphocyte, and monocyte to lymphocyte ratios during anti-TB treatment.

The mean changes from T_0_ to T_end_ of WBC, neutrophil, lymphocyte, monocyte, platelet, MPV, and RDW were −1.93 × 10^3^/μL (95% CI, −2.64 to −1.22), −1.77 × 10^3^/μL (95% CI, −2.44 to −1.09), 0.03 × 10^3^/μL (95% CI, −0.13 to 0.19), −0.05 × 10^3^/μL (95% CI, −0.12 to 0.01), −80.75 × 10^3^/μL (95% CI, −110.80 to −50.71), 0.77 fL (95% CI, 0.24 to 1.30), and −0.41% (95% CI, −1.16 to 0.34). The proportions of subjects that achieved these change for WBC, neutrophil, lymphocyte, monocyte, platelet, MPV, and RDW were 55.1%, 57.1%, 42.8%, 53.5%, 58.6%, 35.7%, and 64.2%. Compared with baseline levels, the WBC, neutrophil, and platelet counts decreased in response to treatment (*P* = 0.0001, *P* = 0.0005, and *P* = 0.001, respectively) (Fig. 3). The decreases in the median lymphocyte count and monocyte count were not statistically significant (Fig. 3). In addition, The mean changes from T_0_ to T_end_ of NLR, PLR, and MLR were −1.34 (95% CI, −1.99 to −0.69), −59.21 (95% CI, −95.90 to −22.52), and −0.04 (95% CI, −0.09 to 0.01). The proportions of subjects that achieved these change for NLR, PLR, and MLR were 67.8%, 60.7%, and 64.2%. The NLR after treatment was significantly decreased compared with baseline (*P = *0.002) (Fig. 4). The PLR and MLR were not significantly changed (Fig. 4).

## Discussion

This study investigated the changes in multiple cytokine and inflammatory markers in 29 patients with active TB following anti-TB drug therapy. We found that the WBC, neutrophil, and platelet counts were decreased following anti-TB drug therapy. The IL-2/IFN-γ ratio and NLR were significantly increased after treatment compared with baseline.

Several exploratory studies have evaluated the potential of cytokine biomarkers other than IFN-γ, particularly TNF-α, IL-2, IL-10, and IL-12, for monitoring anti-TB therapy. TNF-α has been one of the most studied cytokines in previous studies. The largest of these studies, by Eum *et al*., found that TNF-α levels were increased during anti-TB treatment^18^. However, most studies on TB-Ag-stimulated TNF-α responses to anti-TB treatment have consistently reported a reduction in TNF-α levels in whole blood or peripheral blood mononuclear cells during treatment^19,24–26^. Those results were not consistent with the findings of our study. However, our study is different in that these markers were investigated in supernatants collected from the QFT-GIT.

IL-10 is an important anti-inflammatory cytokine reported to affect multiple cell types, such as macrophages, monocytes, dendritic cells, CD4+ T cells, and CD8+ T cells^27^. It inhibits CD4+ T cell responses by inhibiting the antigen-presenting cell function of TB-infected cells^28^. Recently, the secretion of *Mycobacterium tuberculosis* (MTB)-enhanced intracellular survival protein from MTB cells was reported, which possibly increases IL-10 expression^29^. Of the studies that have evaluated the role of IL-10 in TB infection, the findings are not consistent^18,30–32^. The study by Eum *et al*. reported that IL-10 levels increased with treatment^18^. However, another large longitudinal study by Sai Priya *et al*. found a reduction in IL-10 levels in response to treatment^30^. In the current study, we found IL-10 responses to TB specific antigens showed the tendency of decrement following anti-TB therapy. This decrease may be due to modified cytokine expression in infected individuals after treatment.

Several studies have assessed the role of IL-2, which is produced by TB-specific polyfunctional T cells, in monitoring anti-TB treatment responses. Two studies reported lower IL-2 levels at the end compared with the start of treatment^19,33^. Furthermore, Mattos *et al*. reported an increase in IL-2-producing TB-specific T-cells during treatment^34^. In the current study, the changes observed in IL-2 levels were not significant during treatment. However, the IL-2/IFN-γ ratio was significantly increased after treatment. Thus, evaluation of a combination of cytokine biomarkers might be the alternative way to monitor the treatment responses instead of the single cytokine response. Simultaneous measurements of IFN-γ and IL-2 levels help determine T cell cytokine profiles, which reflect the memory phenotype and comprise three main functional T cell subsets: effector cells secreting primarily IFN-γ only, effector memory cells secreting primarily both IFN-γ and IL-2, and central memory cells secreting IL-2 only^35^. Casey *et al*. stated that the proportion of ESAT-6/CFP-10-specific cells secreting IFN-γ only was decreased during treatment. Conversely, the proportion of ESAT-6/CFP-10-specific IFN-γ/IL-2-secreting cells was increased^36^. The increased IL-2/IFN-γ ratio found in our study can be explained by the dynamics of TB-specific T cells.

Several studies have investigated the responses of IL-12 during anti-TB treatment. Eum *et al*. found an increase in IL-12 levels after 2 months of treatment, followed by a decrease to baseline levels after 6 months^18^, whereas Sai Priya *et al*. found a reduction in IL-12 levels over the treatment course^30^. In the current study, the decrease in median IL-12 levels was not statistically significant.

Inflammation plays a significant role in the pathogenesis of pulmonary TB. Recently, the relationships between inflammatory markers, such as neutrophil and platelet counts, and TB have been reported. Several studies demonstrated an important contribution of poly-morphonuclear neutrophils (PMN) to the pathogenesis of TB. Eum *et al*. identified PMNs as the primary immune cell population in sputum and bronchoalveolar lavage (BAL) samples from patients infected with active pulmonary TB^37^. Furthermore, PMN in sputum, BAL, and pulmonary cavity samples carried the greatest mycobacterial loads^37^.

The NLR has emerged as a biomarker of inflammation^38,39^. There are several studies regarding the relationship between chronic obstructive pulmonary disease, TB, or pneumonia and the NLR^38,40–43^. Iliaz *et al*. found a higher NLR in patients with advanced pulmonary TB as opposed to patients with mild to moderate pulmonary TB^38^. Additionally, Abakay *et al*. found reported a significantly higher WBC count, neutrophil count, RDW, and NLR in patients with advanced pulmonary TB compared with mild to moderate pulmonary TB^44^. However, the change in the NLR in TB patients following anti-TB therapy has not been fully evaluated. In the current study, we found that the NLR was significantly decreased after treatment compared with baseline. The NLR is easily determined from routine laboratory tests and is easier and less expensive to measure compared with other inflammatory biomarkers. Therefore, the NLR might be used as a biomarker to evaluate the response to anti-TB treatment.

There are several limitations to this study. First, the number of participants was relatively small, and cases of relapse or treatment failure were not analyzed in this study. Further prospective cohort studies involving larger study samples with variable treatment outcomes are needed. More validation studies are needed in the future which are conducted in other settings. Second, several potential biomarkers such as IP-10, monocyte chemotactic protein 2, and epidermal growth factor were not measured in this study. The studies for other candidate markers or sample types other than Quantiferon supernatants are needed in the future. Third, because of the small numbers of subjects and the absence of a drug resistant cohort, subjects with non-TB pneumonia were not studied as a control group. Further prospective studies involving subjects with non-TB pneumonia are needed.

In conclusion, the current study showed that WBC, neutrophil, and platelet counts were decreased following anti-TB therapy. The IL-2/IFN-γ ratio and the NLR might be potential biomarkers to evaluate the effectiveness of drug therapy in active TB patients.

## Electronic supplementary material

Supplementary Table 1–4
