# Supplementary material for: Changes in cytokine responses to TB antigens ESAT-6, CFP-10 and TB 7.7 and inflammatory markers in peripheral blood during therapy
Source: Sci Rep. 2018 Jan 18;8:1159. doi: 10.1038/s41598-018-19523-7 (PMC5773481; doi:10.1038/s41598-018-19523-7)
Supplement: Supplementary file 1 — Supplementary Table 1–4 [file 41598_2018_19523_MOESM1_ESM.pdf]

Changes in cytokine responses to TB antigens ESAT-6, CFP-10 and TB 7.7 and inflammatory markers in peripheral blood during therapy

Running title: inflammatory and cytokine markers in TB treatment

Ah Young Leem[1*], Joo Han Song[1*], Eun Hye Lee[1], Hyejon Lee[2], Bora Sim[2], Song Yee Kim[1], Kyung Soo Chung[1], Eun Young Kim[1], Ji Ye Jung[1], Moo Suk Park[1], Young Sam Kim[1], Joon Chang[1], Young Ae Kang[1]

[1]Division of Pulmonology, Department of Internal Medicine, Institute of Chest Disease, Severance Hospital, Yonsei University College of Medicine, 50-1 Yonsei-ro, Seodaemun-gu, Seoul 120-752, Republic of Korea

[2]Department of Microbiology and Institute of Immunology and Immunological Disease, Yonsei University College of Medicine, Seoul, Republic of Korea

* These authors contributed equally to this work.

Corresponding Author: Young Ae Kang

Associate Professor

Division of Pulmonology, Department of Internal Medicine, Institute of Chest Disease, Severance Hospital, Yonsei University College of Medicine

50-1 Yonsei-ro, Seodaemun-gu, Seoul 120-752, Republic of Korea

Tel: 82-2-2228-1930, Fax: 82-2-393-6884, E-mail: MDKANG@yuhs.ac

Supplementary Table 1. The changes of levels of IFN-γ, IL-10, IL-12, IL-13, IL-2 and TNF-α in supernatants of QFT-GIT before (T0), after 2 months (T2), and at the end of anti-TB treatment (Tend).

	IFN-γ (TB-Nil)			IL-10 (TB-Nil)			IL-12 (TB-Nil)			IL-13 (TB-Nil)			IL-2 (TB-Nil)			TNF-α (TB-Nil)		
	T0	T2	Tend	T0	T2	Tend	T0	T2	Tend	T0	T2	Tend	T0	T2	Tend	T0	T2	Tend
1	3306.37	1054.45	478.14	-0.09	-2.11	-6.02	32.08	6.44	-6.32	188.61	64.94	96.72	688.6	284.56	134.55	123.44	-27.4	-677.14
2	84.24	39.58	17.79	0.08	-2.35	-2.54	0	0	0	0	-0.86	0	17.78	28.54	24.17	69.97	-490.05	-101.46
3	462.37	78.47	85.38	0.62	-2.99	-14.26	0	0	0	0	0	0	275.16	151.42	157.2	-29.49	304.07	-283.15
4	427.7	277.6	383.01	0.48	-0.16	-0.16	0	0	0	10.4	15.76	13.69	51.42	74.68	102.6	-139.3	-25.33	-110.92
5	1646.57	249.04	688.65	-0.9	-2.02	-0.94	-4.54	0	0	44.97	0	59.93	779.03	148.2	445.79	146.24	-32.11	118.41
6	465.39	10.42	24.66	0.51	-0.87	-1.2	-8.54	0	0	28.12	0	0	123.32	9.43	12.24	-45.27	-177.59	-277.3
7	336.56	300.3	155.66	1.38	-30.05	-4.34	0	-7.5	0	2.83	0	0	257.25	253.88	118.83	77.38	-458.95	-78.84
8	732.14	217.95	275	67.95	8.99	16.84	0	0	0	5.5	0	8.81	177.22	67.21	124	3089.97	388.72	447.52
9	500.11	104.75	71.13	2.55	3.6	-4.47	0	0	0	7.69	3.74	0.86	283.86	101.46	68.44	-22.04	-82.29	-91.39
10	1304.8	586.65	186.73	0.68	2.18	-5.17	0	0	0	58.2	3.74	0	197.24	148.61	58.39	91.56	72.95	736.22
11	367.97	197.76	188.54	-0.32	-2.03	-3.03	0	0	0	-1.88	2.83	0.86	43.69	51.14	49.02	14.86	171.82	-100.12
12	0	595.23	581.75	0	0.09	0.08	0	0	0	0	18.9	30.02	0	244.82	238.02	0	84.8	-83.65
13	6064.24	2502.3	4065.9	1.78	-2.23	-67.68	0	0	2.01	118.68	47.57	151.75	591.6	553.12	431.97	96.57	177.59	-136.83
14	1965.1	613.99	723.18	2.11	-0.31	-0.21	54.37	15.45	68.51	61.24	22.86	47.95	522.43	207.54	258.93	190.36	127.24	-102.16
15	1166.87	388.66	394.91	-6.03	-16.94	1.94	7.33	-1.66	0.74	27.31	11.11	63.23	518.23	279.72	167.21	111.22	-827.79	-170.42
16	172.51	176.13	281.13	-2.19	-44.59	-31.67	-6.96	-5.16	3.39	0.34	7.32	42.14	125.24	127.59	149.64	-62.09	20.91	15.08
17	3961.27	4259.38	1605.8	-0.6	-1.62	-2.06	-2.92	0	0	61.75	127.81	188.52	439.4	812.06	330.93	781.87	145.11	1088.79
18	1225.77	854.7	471.85	3.34	4.09	2.96	-0.41	0	-1.63	143.9	21.57	26.91	499.5	431.32	194.01	640.63	-25.76	36.18
19	1576.52	334.4	1165.13	-29.39	-25.62	-43.52	1.46	14.42	10.21	28.49	0	8.92	218.51	133.35	165.42	173.86	198.63	597.36
20	354.89	683.27	181.74	14.47	-5	16.7	1.66	-2.48	3.31	3.71	30.33	0	93.6	163.32	53.39	237.33	320.74	452.13
21	271.05	90.13	138.8	-0.59	4.34	1.34	2.18	-1.42	5.47	2.8	0	0	111.79	56.51	78.2	213.75	69.05	266.55
22	83.22	376.5	142.22	-4.56	-15.08	-1.26	0	22.25	1.65	0	0	0	50.47	110.95	94.31	141.3	217.13	47.12
23	3272.42	811.19	373.14	-6.22	-13.56	8.62	-3.96	15.09	0	49.07	0.51	11.11	340.53	214.63	65.82	483.18	66.18	168.38
24	328.16	123.42	20.87	0.96	-14.67	8.41	0	2.26	0	0	0	0	74.63	29.91	8.35	338.04	293.51	-161.33
25	87.98	90.43	83.31	-4.85	-19.62	-14.01	32.52	-22.26	-20.31	0	0	2.8	42.86	34.6	49.53	-159.63	-62.25	-70.09
26	208.62	201.56	215.53	-7.12	3.13	-13.92	-3.03	-0.7	6.13	0	0	2.44	5.41	7.43	15.28	-94.61	274.15	164.31
27	2881.07	538.53	439.54	0.17	-3.44	-14.47	8.98	0	9.73	26.92	7.39	10.1	316.53	123.73	106.58	489.02	135.5	204.4
28	2879.82	1010.44	241.22	-0.18	0.52	-15.99	35.18	8.52	7.68	43.4	12.86	-2.47	373.57	203.31	42.43	91.27	44.16	19.51
29	-10.49	38.3	106.38	38.04	-82.91	43.76	0	-3.46	2.57	0	0	0	0	19.61	40.7	91.46	-27.19	109.01

Supplementary Table 2. The changes of IL-2/IFN-γ and IL-10/IFN-γ ratios in supernatant of QFT-GIT during anti-TB treatment.

	IL-10/IFN-γ			IL-2/IFN-γ		
	T0	T2	Tend	T0	T2	Tend
1	0.0000	-0.0020	-0.0126	0.2083	0.2699	0.2814
2	0.0009	-0.0594	-0.1428	0.2111	0.7211	1.3586
3	0.0013	-0.0381	-0.1670	0.5951	1.9297	1.8412
4	0.0011	-0.0006	-0.0004	0.1202	0.2690	0.2679
5	-0.0005	-0.0081	-0.0014	0.4731	0.5951	0.6473
6	0.0011	-0.0835	-0.0487	0.2650	0.9050	0.4964
7	0.0041	-0.1001	-0.0279	0.7644	0.8454	0.7634
8	0.0928	0.0412	0.0612	0.2421	0.3084	0.4509
9	0.0051	0.0344	-0.0628	0.5676	0.9686	0.9622
10	0.0005	0.0037	-0.0277	0.1512	0.2533	0.3127
11	-0.0009	-0.0103	-0.0161	0.1187	0.2586	0.2600
12	0.0000	0.0002	0.0001	0.0000	0.4113	0.4091
13	0.0003	-0.0009	-0.0166	0.0976	0.2210	0.1062
14	0.0011	-0.0005	-0.0003	0.2659	0.3380	0.3580
15	-0.0052	-0.0436	0.0049	0.4441	0.7197	0.4234
16	-0.0127	-0.2532	-0.1127	0.7260	0.7244	0.5323
17	-0.0002	-0.0004	-0.0013	0.1109	0.1907	0.2061
18	0.0027	0.0048	0.0063	0.4075	0.5046	0.4112
19	-0.0186	-0.0766	-0.0374	0.1386	0.3988	0.1420
20	0.0408	-0.0073	0.0919	0.2637	0.2390	0.2938
21	-0.0022	0.0482	0.0097	0.4124	0.6270	0.5634
22	-0.0548	-0.0401	-0.0089	0.6065	0.2947	0.6631
23	-0.0019	-0.0167	0.0231	0.1041	0.2646	0.1764
24	0.0029	-0.1189	0.4030	0.2274	0.2423	0.4001
25	-0.0551	-0.2170	-0.1682	0.4872	0.3826	0.5945
26	-0.0341	0.0155	-0.0646	0.0259	0.0369	0.0709
27	0.0001	-0.0064	-0.0329	0.1099	0.2298	0.2425
28	-0.0001	0.0005	-0.0663	0.1297	0.2012	0.1759
29	-3.6263	-2.1648	0.4114	0.0000	0.5120	0.3826

Supplementary Table 3. The changes of serum inflammatory markers during anti-TB treatment.

	WBC			monocyte			lymphocyte			platelet			RDW			MPV		
	T0	T2	Tend	T0	T2	Tend	T0	T2	Tend	T0	T2	Tend	T0	T2	Tend	T0	T2	Tend
1	5.91	5.43	8.91	0.44	0.36	0.51	1.81	1.46	1.9	205	200	229	12.6	12.5	12.8	9.3	9.3	7.9
2	12.31	15.15	6.53	0.88	0.64	0.38	1.23	1.16	1.55	548	510	371	14.3	15.5	12.7	9.1	6.8	9.8
3	8.38	8.14	6.12	0.42	0.47	0.55	2.22	2.62	2.11	256	230	201	13.9	14.3	14	9.7	7.7	9.5
4	4.24	4.56	3.74	0.28	0.49	0.28	1.15	1.84	1.63	418	258	223	15.6	14.5	14.5	9.1	10.2	10.7
5	9.06	2.88	3.69	0.71	0.22	0.44	1.96	1.27	1.54	436	268	236	14.5	14.4	13.1	7.1	9	9.3
6	5.39	3.69	3.12	0.38	0.28	0.27	1.59	0.64	0.87	250	187	180	13.7	13.7	13	6.9	9.4	9.6
7	6.04	5.26	4.97	0.25	0.4	0.3	1.61	1.73	1.87	181	161	152	12.2	12.7	12.5	8.8	8.3	8.4
8	10.07	4.6	5.44	0.52	0.35	0.22	1.08	1.06	1.32	497	299	290	12.2	15.8	12.4	6.8	8.1	7
9	6.99	6.94	5.84	0.33	0.73	0.62	1.23	1.2	1.66	361	236	250	11.8	12.8	12.6	9.2	7.4	9.5
10	5.5	3.64	4.7	0.38	0.2	0.31	1.6	1.6	1.32	226	233	205	16.7	13.8	15.2	9.7	6.9	9.2
11	7.07	5.39	5.51	0.47	0.31	0.37	1.27	1.34	1.02	193	200	238	12.6	12.8	13.2	9.8	7.4	9.3
12	9.89	5.32	5.68	0.32	0.39	0.38	1.62	1.76	1.65	388	287	274	14.3	15.5	16.9	7.6	10.1	10.7
13	7.2	4.46	4.37	0.07	0.2	0.31	0.88	1.3	1.65	267	207	218	12.4	12.9	12.5	7.7	9.3	9.8
14	6.31	4.95	5.87	0.37	0.36	0.36	1.92	2.47	2.66	269	243	241	12.5	13.5	12.5	7	9	10.2
15	7.1	3.56	4.68	0.36	0.13	0.34	1.77	1.38	1.38	264	249	239	14.6	14.3	16.3	10.7	10.2	10.1
16	5.47	5.53	4.33	0.2	0.26	0.23	1.87	1.62	1.82	251	213	210	11.7	11.9	11.8	8	9.5	10.1
17	10.11	6.63	5.46	0.91	0.48	0.6	2.12	2.52	1.96	325	257	222	13.6	13.9	13.4	7.8	10.7	11.1
18	6.55	3.78	3.35	0.37	0.22	0.25	1.88	2.08	1.6	291	281	244	13.8	14	14.6	9.9	9.7	10.3
19	4.69	5.17	3.16	0.23	0.34	0.18	2.06	1.13	1.27	292	253	251	12.5	12	12	7.2	8.7	8.4
20	8.06	6.74	7.4	0.65	0.71	0.68	1.7	1.75	2.18	250	235	264	13.4	12.4	13.3	10.1	9.9	10
21	6.4	5.65	6.8	0.71	0.47	0.57	2.16	1.2	2.23	290	285	296	13.7	14.1	13.9	9	8.9	8.7
22	11.52	10.89	7.95	0.82	0.72	0.59	2.71	2.82	2.26	408	308	301	13.9	13	12	8.4	8.4	8.5
23	5.75	6.6	3.94	0.31	0.39	0.43	1.58	1.97	1.38	360	255	209	12.4	13.1	12.1	10	9.7	9.9
24	7.64	4.47	5.68	0.39	0.39	0.46	1.93	1.93	2.76	426	222	193	19.3	12.3	11.7	7.8	8.1	8.1
25	5.98	4.43	5.89	0.49	0.49	0.4	0.78	0.84	0.78	393	306	307	12.6	13.2	12.2	7.4	9.5	10.1
26	5.64	4.1	3.46	0.33	0.4	0.33	1.51	1.09	1.5	393	221	160	11.8	12	11.8	8.9	8.7	9
27	4.92	3.88	4.03	0.64	0.59	0.37	1.4	1.54	1.95	269	227	238	16.5	12.2	12.2	10.2	10.4	10.3
28	5.56	5.58	4.94	0.49	0.3	0.35	2.83	1.36	2.54	215	179	170	15.2	15.7	17.6	10.6	10	10
29	8.43	3.99	6.6	-	-	-	-	-	-	320	274	288	-	-	-	-	-	-

Supplementary Table 4. The changes of neutrophil to lymphocyte, platelet to lymphocyte, and monocyte to lymphocyte ratios during anti-TB treatment.

	MLR			PLR			NLR		
	T0	T2	Tend	T0	T2	Tend	T0	T2	T6
1	0.2431	0.2466	0.2684	113.2597	136.9863	120.5263	1.9006	2.3425	3.2263
2	0.7154	0.5517	0.2452	445.5284	439.6552	239.3548	8.2602	11.3276	2.7871
3	0.1892	0.1794	0.2607	115.3153	87.7863	95.2607	2.4820	1.7099	1.6161
4	0.2435	0.2663	0.1718	363.4783	140.2174	136.8098	2.3217	1.0870	1.0307
5	0.3622	0.1732	0.2857	222.4490	211.0236	153.2467	2.9898	1.0157	1.0649
6	0.2390	0.4375	0.3103	157.2327	292.1875	206.8965	1.7736	3.7813	1.8736
7	0.1553	0.2312	0.1604	112.4224	93.0636	80.8941	2.5528	1.7168	1.4529
8	0.4815	0.3302	0.1667	460.1852	282.0755	219.6970	7.6944	2.9057	2.7727
9	0.2683	0.6083	0.3735	293.4959	196.6667	150.6024	4.2846	4.1000	2.0663
10	0.2375	0.1250	0.2348	141.2500	145.6250	155.3030	2.0313	1.0188	2.2500
11	0.3701	0.2313	0.3627	151.9685	149.2537	233.3333	4.1575	2.6716	3.9902
12	0.1975	0.2216	0.2303	239.5062	163.0682	166.0606	4.7160	1.7045	2.0727
13	0.0795	0.1538	0.1879	303.4091	159.2308	132.1212	7.0000	2.1923	1.4242
14	0.1927	0.1457	0.1353	140.1042	98.3806	90.6015	1.9635	0.7854	1.0000
15	0.2034	0.0942	0.2464	149.1525	180.4348	173.1884	2.7684	1.3333	2.0652
16	0.1070	0.1605	0.1264	134.2246	131.4815	115.3846	1.6898	2.0309	1.1648
17	0.4292	0.1905	0.3061	153.3019	101.9841	113.2653	3.1415	1.3770	1.3929
18	0.1968	0.1058	0.1563	154.7872	135.0962	152.5000	2.2181	0.6346	0.8688
19	0.1117	0.3009	0.1417	141.7476	223.8938	197.6378	0.9417	3.0177	1.1969
20	0.3824	0.4057	0.3119	147.0588	134.2857	121.1009	2.9765	2.1829	1.9174
21	0.3287	0.3917	0.2556	134.2593	237.5000	132.7354	1.5417	3.1833	1.7220
22	0.3026	0.2553	0.2611	150.5535	109.2199	133.1858	2.8819	2.5496	2.1549
23	0.1962	0.1980	0.3116	227.8481	129.4416	151.4493	2.3481	2.0863	1.4348
24	0.2021	0.2021	0.1667	203.6269	158.5492	111.2319	1.7617	1.0363	0.9348
25	0.6282	0.5833	0.5128	503.8462	263.0952	205.1282	5.2821	3.0476	2.2436
26	0.2185	0.3670	0.2200	178.1457	208.2569	158.6667	1.8278	2.0183	1.3600
27	0.4571	0.3831	0.1897	153.5714	116.2338	87.1795	2.2500	2.0519	1.2718
28	0.1731	0.2206	0.1378	113.0742	201.4706	113.3858	1.7845	1.6765	1.4370
